# CD4^+^ T Follicular Helper and IgA^+^ B Cell Numbers in Gut Biopsies from HIV-Infected Subjects on Antiretroviral Therapy Are Similar to HIV-Uninfected Individuals

**DOI:** 10.3389/fimmu.2016.00438

**Published:** 2016-10-24

**Authors:** John Zaunders, Mark Danta, Michelle Bailey, Gerald Mak, Katherine Marks, Nabila Seddiki, Yin Xu, David J. Templeton, David A. Cooper, Mark A. Boyd, Anthony D. Kelleher, Kersten K. Koelsch

**Affiliations:** ^1^St Vincent’s Centre for Applied Medical Research, St Vincent’s Hospital, Sydney, NSW, Australia; ^2^The Kirby Institute, The University of New South Wales, Sydney, NSW, Australia; ^3^St Vincent’s Hospital, Clinical School, Sydney, NSW, Australia; ^4^Equipe 16, INSERM U955, Créteil, France; ^5^Faculté de médecine, Université Paris Est, Créteil, France; ^6^Vaccine Research Institute (VRI), Créteil, France; ^7^RPA Sexual Health, Royal Prince Alfred Hospital, Sydney, NSW, Australia

**Keywords:** HIV, gut-associated lymphoid tissue, CD4^+^ T lymphocytes, T follicular helper cells, germinal centers, IgA B cells

## Abstract

**Background:**

Disruption of gastrointestinal tract epithelial and immune barriers contribute to microbial translocation, systemic inflammation, and progression of HIV-1 infection. Antiretroviral therapy (ART) may lead to reconstitution of CD4^+^ T cells in gut-associated lymphoid tissue (GALT), but its impact on humoral immunity within GALT is unclear. Therefore, we studied CD4^+^ subsets, including T follicular helper cells (Tfh), as well as resident B cells that have switched to IgA production, in gut biopsies, from HIV^+^ subjects on suppressive ART compared to HIV-negative controls (HNC).

**Methods:**

Twenty-three HIV^+^ subjects on ART and 22 HNC undergoing colonoscopy were recruited to the study. Single-cell suspensions were prepared from biopsies from left colon (LC), right colon (RC), and terminal ileum (TI). T and B lymphocyte subsets, as well as EpCAM^+^ epithelial cells, were accurately enumerated by flow cytometry, using counting beads.

**Results:**

No significant differences in the number of recovered epithelial cells were observed between the two subject groups. However, the median TI CD4^+^ T cell count/10^6^ epithelial cells was 2.4-fold lower in HIV^+^ subjects versus HNC (19,679 versus 47,504 cells; *p* = 0.02). Similarly, median LC CD4^+^ T cell counts were reduced in HIV^+^ subjects (8,358 versus 18,577; *p* = 0.03) but were not reduced in RC. Importantly, we found no significant differences in Tfh or IgA^+^ B cell counts at either site between HIV^+^ subjects and HNC. Further analysis showed no difference in CD4^+^, Tfh, or IgA^+^ B cell counts between subjects who commenced ART in primary compared to chronic HIV-1 infection. Despite the decrease in total CD4 T cells, we could not identify a selective decrease of other key subsets of CD4^+^ T cells, including CCR5^+^ cells, CD127^+^ long-term memory cells, CD103^+^ tissue-resident cells, or CD161^+^ cells (surrogate marker for Th17), but there was a slight increase in the proportion of T regulatory cells.

**Conclusion:**

While there were lower absolute CD4^+^ counts in the TI and LC in HIV^+^ subjects on ART, they were not associated with significantly reduced Tfh cell counts or IgA^+^ B cells, suggesting that this important vanguard of adaptive immune defense against luminal microbial products is normalized following ART.

## Introduction

Several studies have focused on the effect of primary HIV-1 infection (PHI) on CD4^+^ T cells in gut-associated lymphoid tissue (GALT) as a defining event in the pathogenesis of eventual chronic HIV-1 infection (CHI) [reviewed in Ref. (1, 2)]. GALT is widely believed to contain the majority of CD4^+^ T cells in the body, and PHI is believed to result in massive depletion of CD4^+^ T cells in the lamina propria (3–5), compared to the slower decline of CD4^+^ T cell counts in blood (6–8). In particular, the chronic T cell activation that is characteristic of untreated CHI may be due to damage to the epithelial barrier in GALT, leading to systemic translocation of microbial products into lymphoid tissues (2, 8–10), which in turn may lead to further systemic activation of CD4^+^ T cells and their continued depletion.

Potent antiretroviral therapy (ART), even when fully suppressive for years, may not necessarily lead to full restoration of normal numbers of CD4^+^ T cells in GALT (11–13), and even commencing ART during PHI may not completely restore CD4^+^ T cells in GALT (11), unless subjects are treated during the very earliest stages of infection (14). This dysregulation of GALT may also lead to its role as a continuing reservoir for HIV-1 replication, despite ART (15, 16).

Most studies of CD4^+^ T cell depletion in GALT during HIV infection have particularly looked at a deficit in Th17 cells, since these are believed to contribute to epithelial cell barrier integrity (7, 10, 14, 17, 18). It has been reported that a large majority of CD4^+^ T lymphocytes in human GALT express CCR5 (19, 20) and that many of these cells are highly activated (21), making this sub-population of CD4^+^ T cells highly susceptible to HIV-1 infection and depletion. Furthermore, it has been reported that T regulatory cells (Tregs) are significantly increased in tonsil tissue during HIV-1 infection (22) and also in GALT during untreated, but not treated, HIV-1 infection (23). Altogether, it is believed that normal CD4 T cell functions in GALT are significantly compromised in HIV-1 infection and may not be completely normalized by ART.

However, prior to reaching the epithelial cells, gut microbes must first penetrate the mucous layer in the lumen, and the front-line adaptive immune defense in this layer is IgA, produced locally and secreted into the lumen (24, 25). This IgA production in turn is largely dependent on CD4^+^ T cell help, mediated by T follicular helper (Tfh) cells in local germinal centers (GCs) (24).

Since large-scale depletion of CD4^+^ T cells has been reported in GALT, it is probable that Tfh are also depleted, and in fact, it has been reported that GCs in GALT have abnormally high levels of apoptosis during PHI (26) and GC may not normalize after ART (18). Furthermore, murine studies have suggested that gut Tfh develop from Th17 precursors (27), and Th17 cells are reportedly also lost very early in HIV-1 infection (7, 14, 18). We, and others, have recently found that Tfh cells in GCs, peripheral lymph nodes, and spleen are highly infected in both SIV and HIV infections (28–32). Taken together, we hypothesized that Tfh, along with other CD4^+^ T cell subsets in GALT, would be depleted early in HIV-1 infection and may not be fully reconstituted during ART, which would in turn lead to a deficit of IgA^+^ B cells, further compromising the integrity of the epithelial barrier.

We aimed to identify and quantify the Tfh subset of CD4^+^ T cells, in gut biopsies from subjects on fully suppressive ART and also IgA^+^ B cells present in the same samples. At the same time, in comparison to the effect on Tfh, we also studied the effect of treated HIV-1 infection on other key subsets of CD4^+^ T cells, including CCR5^+^ memory CD4^+^ T cells, CD127^+^ long-term memory cells, CD103^+^ tissue-resident memory T (Trm) cells, CD161^+^ cells, which is a surrogate cell surface maker for Th17 cells in human GALT (33), and CD25^+^CD127^dim^ Tregs.

In addition to studying T lymphocyte subsets, we employed polychromatic flow cytometry to enumerate epithelial cells in the same single cell suspensions, using the cell surface marker EpCAM (CD326) (34). It has recently been suggested that proliferation of gut epithelial cells is increased in CHI (35). In order to measure proliferating cells we measured Ki-67 expression in EpCAM^+^ cells by flow cytometry.

## Materials and Methods

### Subjects and Biopsy Collection

Twenty-two HIV-uninfected healthy adult volunteers and 23 HIV^+^ subjects on fully suppressive ART underwent colonoscopy to provide biopsy specimens, through the endoscopy unit at St Vincent’s Hospital, Sydney. The study protocols were approved by the Local Ethics Committee (references HREC 8/SVH/20 and HREC 11/SVH/193), and written informed consent was obtained from each participant. Subject demographics are shown in Table S1 in Supplementary Material.

Endoscopic biopsies from the left colon (LC), right colon (RC), and terminal ileum (TI) were obtained during colonoscopy, with all biopsies taken from standardized sites, as previously described (36).

### Biopsy Processing

Ten pinch biopsies taken from each site were placed in RPMI medium (Life Technologies) containing 10% fetal calf serum (FCS) (Bovogen, VIC, Australia), as well as 100 U of penicillin/streptomycin (Life Technologies; R10/P/S) to minimize bacterial contamination from the GALT samples (37). Biopsies were washed three times with HBSS (Life Technologies) containing 1% PenStrep, 0.1M DTT (Life Technologies) (pH 8.0), and 0.15% EDTA (Sigma-Aldrich, MO, USA) (0.5M) (38) on a shaking incubator at 130 rpm at 37°C for 20 min. Supernatants collected after each wash step were retained for each site. Biopsies were then minced using sterile scissors and enzymatically digested with 200 U/ml collagenase type III (Sigma-Aldrich) (37–39) and 200 mg/ml DNase (Sigma-Aldrich) in R10/P/S for 1 h at 37°C in a shaking incubator at 110 rpm. A single-cell suspension was prepared by pushing digested tissue through a 70-μm cell strainer for flow cytometry.

### Monoclonal Antibodies

Fluorochrome-conjugated monoclonal antibodies (mAbs) used in this study were CD45-PerCP and -Alexa Fluor 700, EpCam-FITC and -BV510, CD3-PerCP-Cy5.5, CD4-PE, -PE-Cy7, -BV786, and -AlexaFluor 700, CD8-APC-Cy7, CD45RA-BV605, HLA-DR-FITC, and -APC-Cy7, CD38-APC and -PE-Cy7, CD25-APC and -BB515, CD127-PE-CF594, CD103-PE, CXCR5-Alexa Fluor 647, CD16-BV421, and CD56-APC from BD Biosciences (San Jose, CA, USA); CD4^+^ 5RA-ECD (clone 2H4) from Beckman Coulter (Hialeah, FL, USA); CD127-eFluor450 and CD62L-APC-eFluor780 from eBioscience (San Diego, CA, USA); PD-1 (PE or APC, clone EH-12) and CD127-BV421 and -PE-Cy7 from BioLegend (San Diego, CA, USA); and IgA-PE and CD161-APC and -PE-Cy7 (Miltenyi Biotec, Germany). All antibodies were used according to the manufacturers’ directions.

For detection of surface markers, gut biopsy cells were stained with fluorochrome-conjugated antibodies for 15 min at room temperature, washed once with PBA [Dulbecco’s phosphate-buffered saline (DPBS) containing 0.5% BSA and 0.1% sodium azide], and resuspended in 0.5% paraformaldehyde (Electron Microscopy Sciences, PA, USA) in DPBS (PFA) for fixation, as previously described for human immunophenotyping (40). Intracellular staining for Ki-67 was performed as previously described (40), and intracellular staining for Bcl-6 was performed as previously described (31).

### CCR5 Staining

An indirect immunofluorescence staining protocol (41) was optimized for detection of CCR5 (31, 42). Gut biopsy cells were stained with 10 μg/ml purified mAb against CCR5 (clone 2D7; BD Biosciences) for 15 min at RT, washed once with PBA, and incubated for 15 min at RT with a 1:50 dilution of PE-conjugated F(ab′)_2_ fragment goat anti-mouse IgG (H + L chain-specific) PE (human, bovine, rabbit, swine serum protein absorbed) secondary antibody (Jackson ImmunoResearch Laboratories, West Grove, PA, USA). Cells were then washed twice with PBA, blocked with 10% mouse serum for 10 min at room temperature, and stained with monoclonal antibodies to other surface proteins as described above. A negative control was performed in parallel, in which the primary anti-CCR5 mAb was not added.

### Flow Cytometry

**Figure 1:**
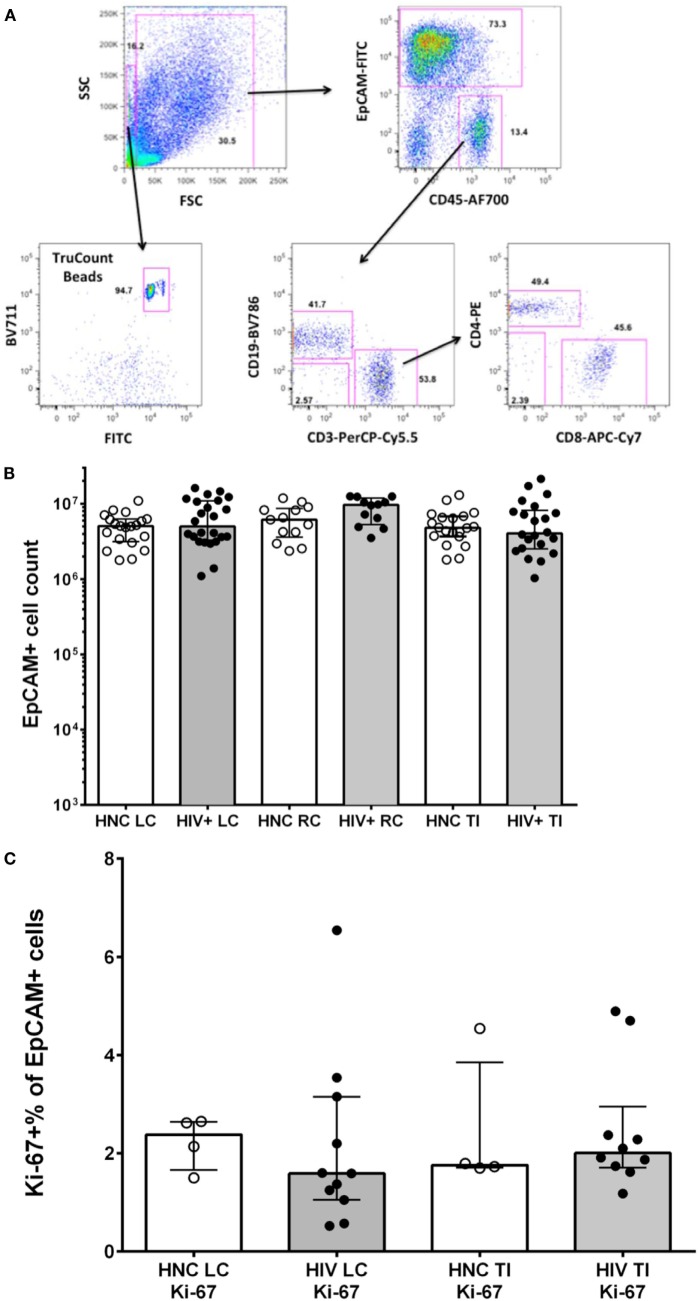
**(A)** Sequential gating strategy to count lymphocytes and epithelial cells in gut biopsy specimens is shown in representative flow plots for an HIV-negative control adult terminal ileum biopsy. **(B)** Epithelial cell counts from HIV-negative controls (HNC) and HIV^+^ subjects on ART, respectively, for left colon (LC), right colon (RC), and terminal ileum (TI). **(C)** Proliferation rates of epithelial cells, measured as Ki-67^+^ % of EpCAM^+^ epithelial cells, in HIV-negative controls (HNC) compared with HIV^+^ subjects on ART.

Stained gut biopsy cells were analyzed on a four-laser BD LSR II with FACSDiva 6.0 software (BD Biosciences) as previously described (40, 43). Cells were gated first on a broad forward versus side scatter gate, then on a CD45 versus EpCAM plot to remove debris and epithelial cells, and define CD45^+^EpCAM^−^ hematopoietic cells (Figure 1A). CD3 was used to define T lymphocytes in the CD45^+^ gate, which were then gated on CD4^+^ and CD8 and subsequently on various subsets as shown. CD19 was used to define B lymphocytes in the CD45^+^ gate, which were then gated on various subsets as indicated.

Cell counts, including CD4^+^ lymphocytes, in the single cell suspensions were determined by adding 100 μl of the single cell suspension preparation sample to mAbs in a TruCount tube (BD Biosciences), incubating for 15 min at RT, and fixing with 0.5-ml PFA, before analyzing by flow cytometry, as above. The number of cells in the 100-μl sample was calculated according to the manufacturer’s directions, and the total number of CD4^+^ T cells recovered was then determined.

### Statistical Analysis

Data are shown as medians and interquartile ranges. Statistical significance between subject groups was analyzed by Mann–Whitney test, with *p* < 0.05 considered statistically significant (PRISM 6, GraphPad Software, CA, USA). The correlation between Tfh cell numbers and IgA^+^ was analyzed by non-parametric Spearman correlation (PRISM 6). Results for four patients who commenced therapy during PHI (36) were compared to all other HIV^+^ subjects on ART by Mann–Whitney test.

## Results

### Epithelial Cell Counting in Gut Biopsies

We used a polychromatic flow cytometry strategy of initial gating based on EpCAM staining of epithelial cells versus CD45 staining of lymphocytes, to accurately count both CD45^+^ lymphocytes and EpCAM^+^ epithelial cells in the single cell suspensions, using counting beads (Figure 1A).

The results show that the number of epithelial cells counted in biopsies was consistent between subject groups (Figure 1B). Minor variations in epithelial cell counts are in line with slight effects of variability in the amount of tissue obtained between specimens or in the efficiency of digestion between samples. The number of epithelial cells in each cell suspension was then used to normalize comparisons of lymphocyte counts between specimens (see below).

Since it has recently been suggested that proliferation of gut epithelial cells is increased in CHI (35), we measured Ki-67 expression in EpCAM^+^ cells by flow cytometry. We found relatively low levels, with medians of 1.5–2.5% of epithelial cells that were Ki-67^+^, but no significant differences between subject groups (Figure 1C).

### Assessing CD4^+^ and CD8^+^ T Cell Numbers

Virtually, all CD45^+^ cells were CD3^+^ T lymphocytes and CD19^+^ B lymphocytes (Figure 1A, see lower middle flow plot), with very few cells identified as neutrophils (CD16^+^, side scatter high, CD45^dim^) or NK cells (CD56^+^CD45^high^) (data not shown). From these CD45^+^ lymphocyte populations, CD4^+^ T cell, CD8^+^ T cells, and B cell counts, respectively, were obtained from the TruCount bead counts (Figure 1A), and calculated as the number of each subset per 10^6^ epithelial cells.

**Figure 2:**
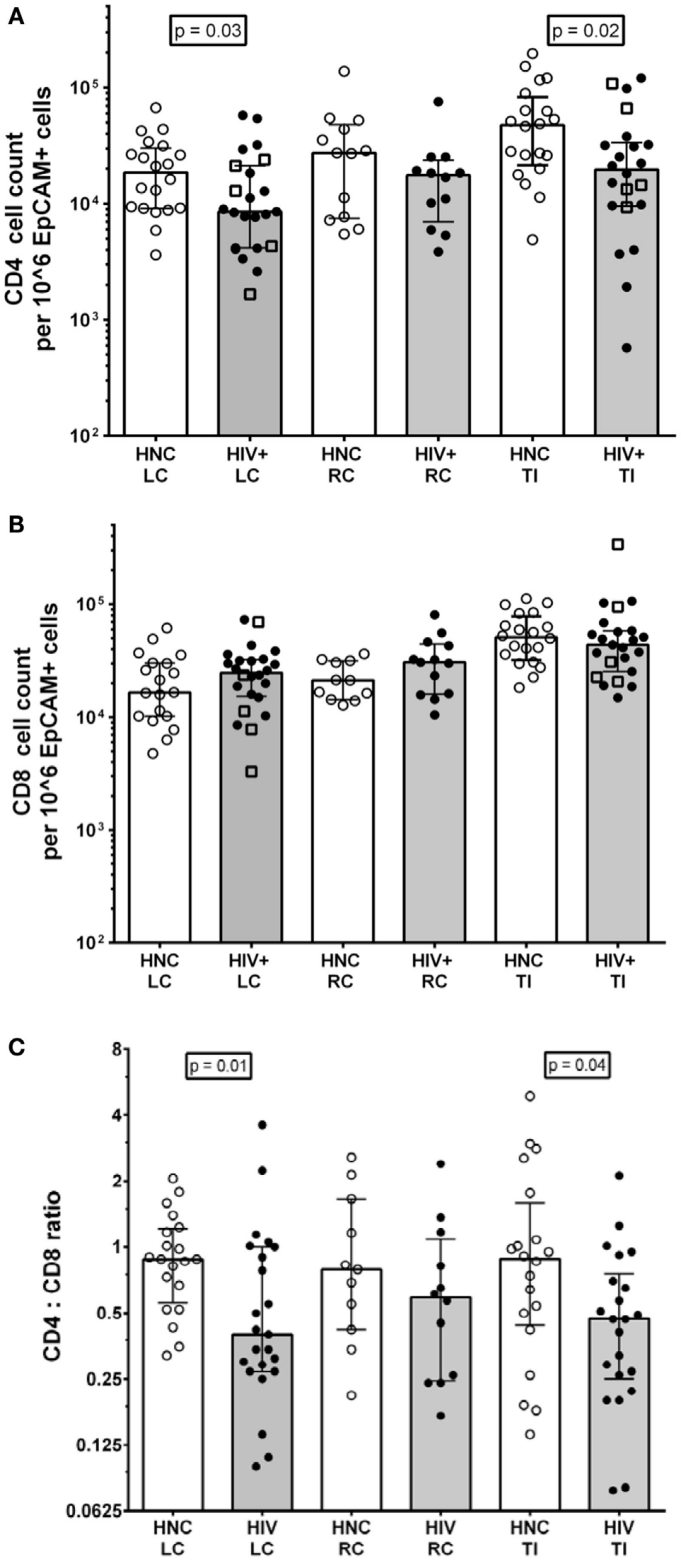
**(A)** CD4^+^ cell counts from gut biopsy single-cell suspensions from HIV-negative controls (HNC) compared with HIV^+^ subjects on ART, respectively, for left colon (LC), right colon (RC), and terminal ileum (TI). Open square symbols indicate HIV^+^ subjects who commenced ART during primary HIV-1 infection. **(B)** CD8^+^ cell counts from gut biopsy single cell suspensions from HIV-negative controls (HNC) compared with HIV^+^ subjects on ART, respectively, for left colon (LC), right colon (RC), and terminal ileum (TI). **(C)** CD4:CD8 ratios HIV-negative controls (HNC) compared with HIV^+^ subjects on ART, respectively, for left colon (LC), right colon (RC), and terminal ileum (TI).

The results show that there were just over twofold reductions in the CD4^+^ T cell counts from LC and TI biopsies from HIV^+^ subjects on ART compared to HIV-negative controls (HNC), respectively (Figure 2A), but no significant difference was seen for RC biopsies. Included in the HIV^+^ group were five subjects who commenced ART during PHI, as part of the PINT trial (36, 44), and their LC and TI biopsies CD4^+^ T cell counts did not differ significantly from the other HIV^+^ subjects (Figure 2A).

There were no significant differences in CD8 T cell count (Figure 2B), but there were significant decreases in the CD4:CD8 ratios in biopsies from LC and TI from the HIV^+^ subjects, compared to healthy adult controls (Figure 2C). The CD4:CD8 ratios in the biopsies from HIV^+^ subjects were lower than the corresponding median ratio of 0.94 in peripheral blood (Table S1 in Supplementary Material).

### T Follicular Helper CD4^+^ T Cells, Germinal Center B Cells, and IgA^+^ B Cells in Gut Biopsies

**Figure 3:**
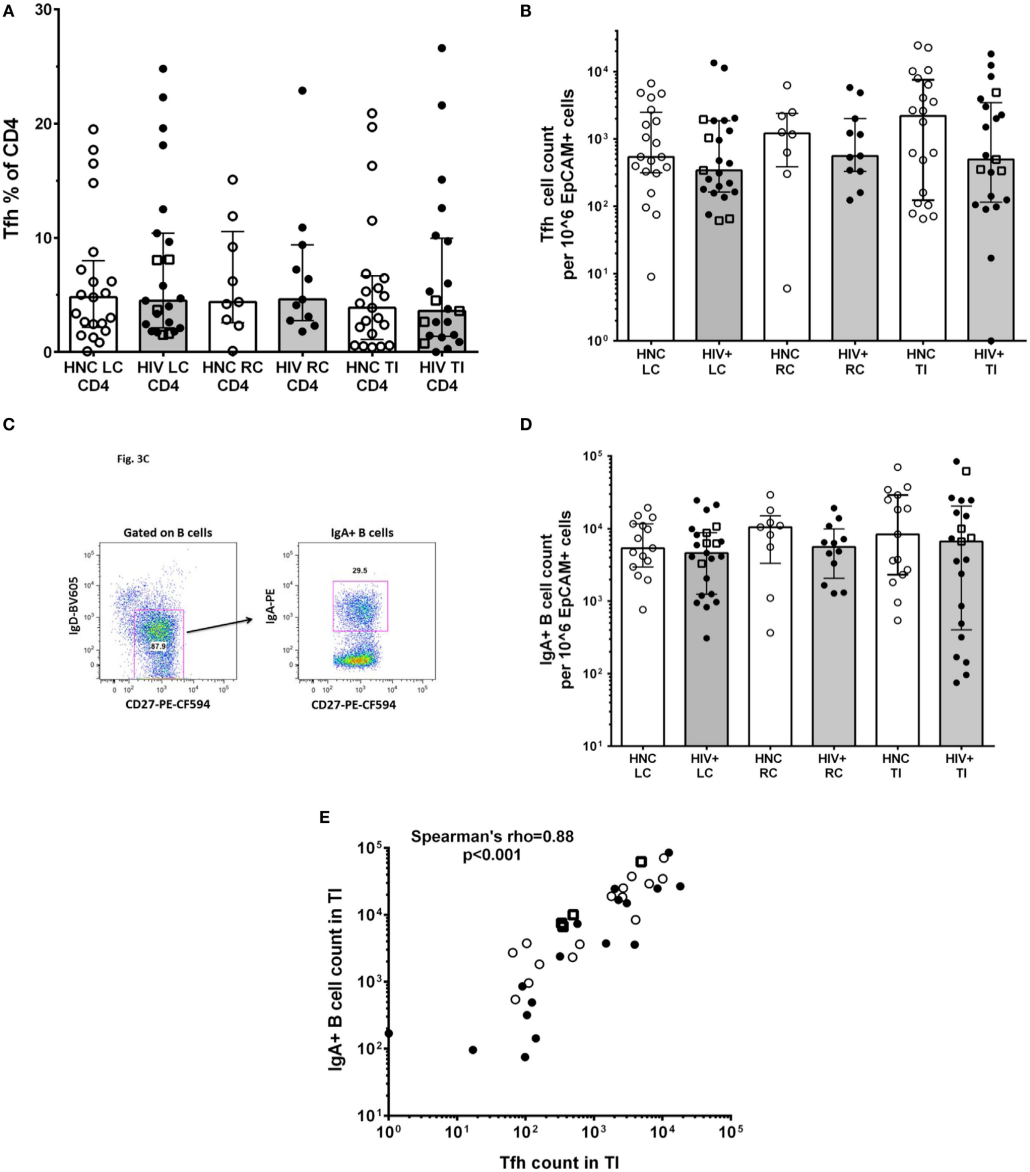
**(A)** PD-1^high^CXCR5^+^ Tfh cells % of CD4^+^ in gut biopsies from HIV-negative controls (HNC) compared with HIV^+^ subjects on ART, respectively, for left colon (LC), right colon (RC), and terminal ileum (TI). **(B)** Tfh cell counts in gut biopsies from HIV-negative controls (HNC) compared with HIV^+^ subjects on ART, respectively, for left colon (LC), right colon (RC), and terminal ileum (TI). Open square symbols indicate HIV^+^ subjects who commenced ART during primary HIV-1 infection. **(C)** Representative flow plots showing gating for IgA^+^CD27^+^ B cells in gut biopsies. **(D)** IgA^+^CD27^+^ B cell counts in gut biopsies from HIV-negative controls (HNC) compared with HIV^+^ subjects on ART, respectively, for left colon (LC), right colon (RC), and terminal ileum (TI). Open square symbols indicate HIV^+^ subjects who commenced ART during primary HIV-1 infection. **(E)** Correlation between IgA^+^ B cell counts in terminal ileum (TI) and Tfh cell counts in terminal ileum (TI), for each individual subject. Open square symbols indicate HIV^+^ subjects who commenced ART during primary HIV-1 infection.

An important function of CD4^+^ T lymphocytes in GALT is to provide help to local B cells to switch to IgA antibody production, which is then secreted into the lumen. IgA and the mucous layer provide first-line protection against pathogenic microbes and are integral to gut microbiome homeostasis (24, 25). This CD4^+^ T cell help to B cells is provided by a specialized subset of Tfh cells, which we identified in the gut biopsies as CD45RA^neg^PD-1^high^ CXCR5^+^ (31); these cells also expressed the Tfh-lineage-specific transcription factor Bcl-6 (Figure S1 in Supplementary Material). The results show that there were no significant differences in the proportion of CD4^+^ T cells in gut biopsies that were Tfh (Figure 3A) between HIV^+^ and HNC subjects. Furthermore, there were no significant differences in Tfh cell counts in gut biopsy samples from HIV^+^ subjects on ART compared to healthy adult controls (Figure 3B).

We could also identify GC B cells in the gut biopsy samples as CD19^+^CD20^high^CD38^high^Ki-67^+^ cells (Figure S2 in Supplementary Material), which is consistent with the detection of Tfh cells in the same samples.

IgA^+^ B cells in the gut biopsy samples were identified as CD19^+^IgD^−^CD27^+^IgA^+^ lymphocytes (Figure 3C). The overall results show that there was no significant difference in the number of IgA^+^ B cells in gut biopsy samples from HIV^+^ subjects on ART compared to healthy adult controls (Figure 3D). Importantly, there was a highly significant correlation between the number of Tfh cells and the number of IgA^+^ B cells in TI biopsies (Figure 3E; Spearman’s rho = 0.89; *p* < 0.001).

### CCR5^+^CD4^+^ T Cells

**Figure 4:**
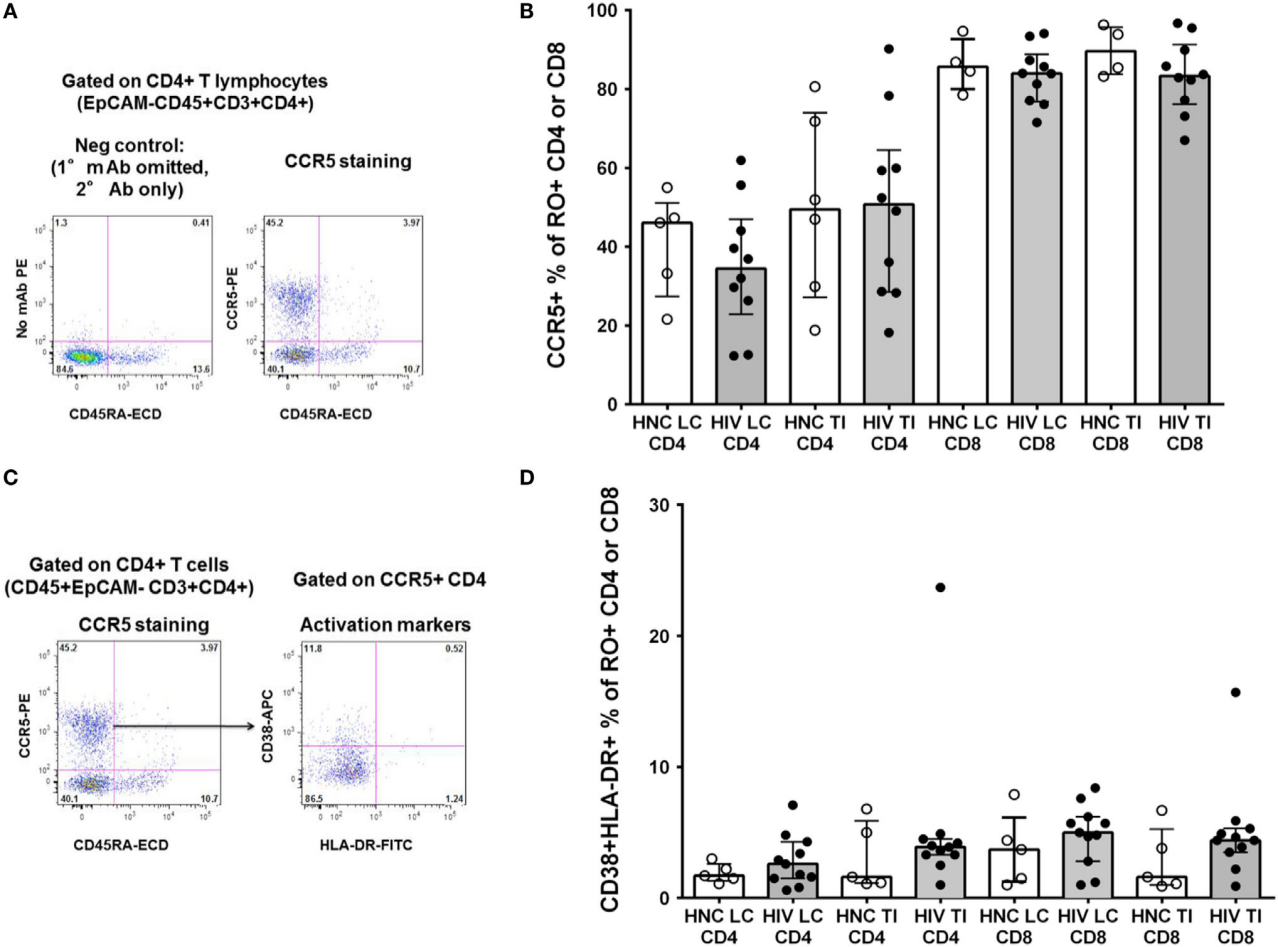
**(A)** Indirect immunofluorescence to measure CCR5^+^CD4^+^ T cells. Representative flow plot on left shows negative control where anti-CCR5 primary monoclonal antibody was omitted. Flow plot on right shows CCR5 expression on memory (CD45RA^−^) CD4^+^ T cells from a TI biopsy from a healthy control (HNC). **(B)** CCR5^+^ % of memory CD4^+^ and CCR5^+^ % of memory CD45RO^+^CD8^+^ T cells in gut biopsies from HIV-negative controls (HNC) compared with HIV^+^ subjects on ART, respectively, for left colon (LC) and terminal ileum (TI). **(C)** Representative flow plot on right shows co-expression of CD38 and HLA-DR on CCR5^+^ memory CD4^+^ T cells (gated on left, from **(A)**, above) from a TI biopsy from a healthy control (HNC). **(D)** Co-expression of CD38 and HLA-DR on CCR5^+^ memory CD4^+^ and CCR5^+^ memory CD8^+^ T cells, in gut biopsies from HIV-negative controls (HNC) compared with HIV^+^ subjects on ART, respectively, for left colon (LC) and terminal ileum (TI).

We also studied other important subsets of CD4^+^ T cells in the gut biopsies that may have been selectively depleted. We have recently optimized staining for CCR5 on CD4^+^ T lymphocytes in non-human primate lymphoid tissue (31) and human blood (42). Therefore, we re-evaluated CCR5 expression using the optimized staining protocol (representative flow plots are shown in Figure 4A). Overall, approximately half of CD4^+^ T cells in the gut biopsies were CCR5^+^ (Figure 4B). We found no significant difference in the proportion of CD4^+^ T cells that were CCR5^+^ between HIV^+^ subjects and HNC (Figure 4B), suggesting that there was no selective depletion of the CCR5^+^ subset, and this did not account for the difference in CD4^+^ T cell counts.

In contrast to the CD4^+^ T cells, a much higher proportion of CD8^+^ T cells were also CCR5^+^, around 90%, from both subject groups (Figure 4B).

Also, we gated on the CCR5^+^CD4^+^ T cells in gut biopsies from healthy adult controls and studied co-expression of the activation markers CD38 and HLA-DR (representative flow data is shown in Figure 4C). Overall, we found that in HNC, <10% of either CCR5^+^CD4^+^ or CCR5^+^CD8^+^ T cells exhibited co-expression of CD38 and HLA-DR (Figure 4D), and there were no significant differences compared to HIV^+^ subjects.

#### Other Subsets of Memory CD4^+^ T Lymphocytes

**Figure 5:**
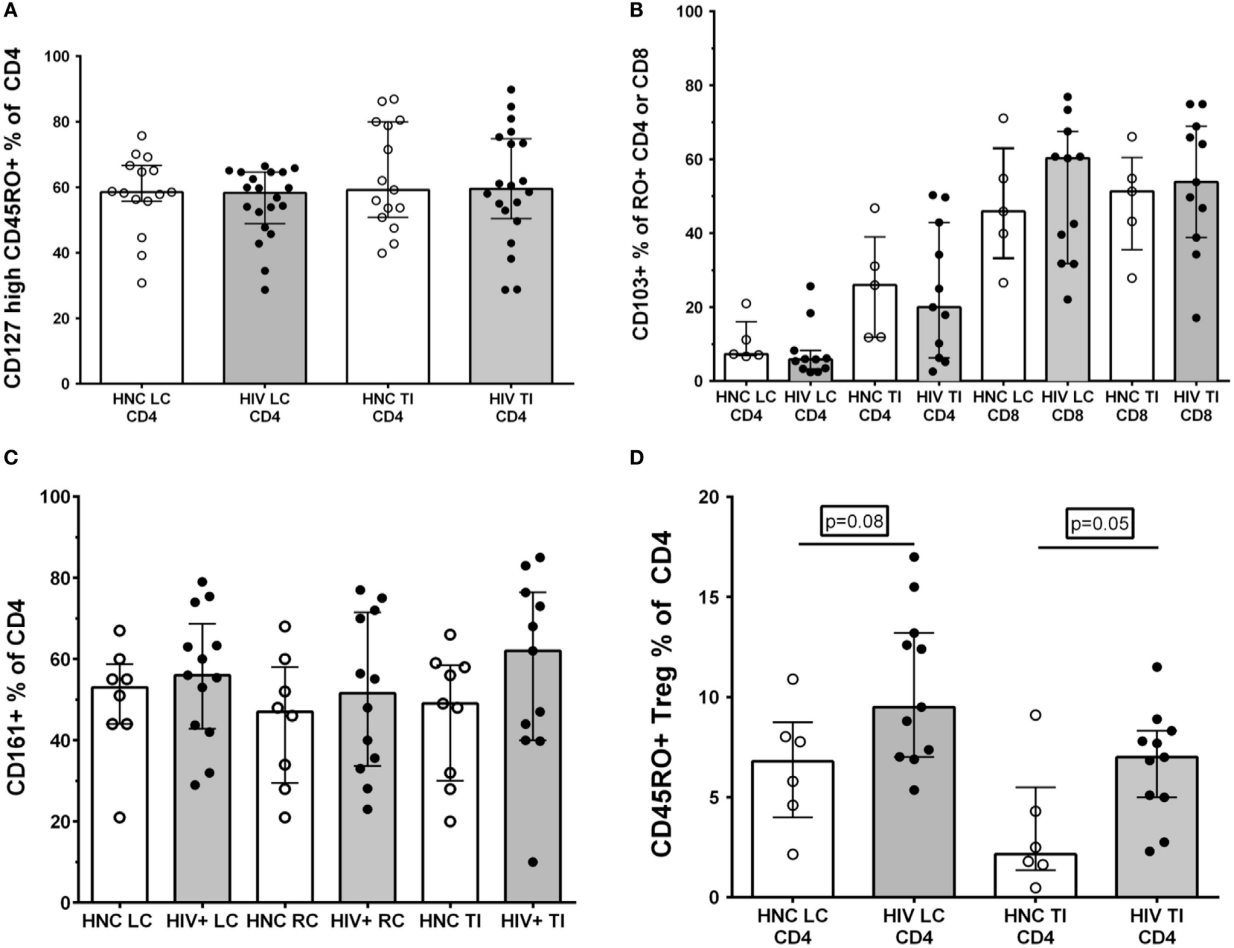
**(A)** CD127^high^ % of memory CD45RO^+^CD4^+^ T cells in gut biopsies from HIV-negative controls (HNC) compared with HIV^+^ subjects on ART, respectively, for left colon (LC) and terminal ileum (TI). **(B)** CD103^+^ % of memory CD45RO^+^CD4^+^ and CD103^+^ % of memory CD45RO^+^CD8^+^ T cells in gut biopsies from HIV-negative controls (HNC) compared with HIV^+^ subjects on ART, respectively, for left colon (LC) and terminal ileum (TI). **(C)** CD161^+^ % of CD4^+^ T cells in gut biopsies from HIV-negative controls (HNC) compared with HIV^+^ subjects on ART, respectively, for left colon (LC) and terminal ileum (TI). **(D)** CD25^high^CD127^dim^CD45RO^+^ Tregs % of CD4^+^ T cells in gut biopsies from HIV-negative controls (HNC) compared with HIV^+^ subjects on ART, respectively, for left colon (LC) and terminal ileum (TI).

We and others have found that the largest proportional decline in CD4^+^ T cells in blood during early HIV-1 infection is associated with loss of memory CD4^+^ T cells that express the alpha chain of the IL-7 receptor, CD127 (43, 45, 46), with eventual loss of IL-7 homeostasis in late-stage disease (47). Therefore, we analyzed the CD127^+^ subset as a percentage of memory CD4^+^ T cells in gut biopsy samples, but found no significant differences between HIV^+^ subjects and HNC (Figure 5A).

Second, we differentiated between CD103^+^ intraepithelial, tissue-resident CD4^+^ T cells (48) versus transient CD103^−^CD4^+^ T cells. Here, we found that only a very small minority of CD4^+^ T cells in LC biopsies were CD103^+^, but a larger proportion, around 20% of CD4^+^ T cells, were CD103^+^ in TI biopsies (Figure 5B). However, again, we found no significant differences in CD103^+^CD4^+^ T cells between biopsies from HIV^+^ subjects and HNC (Figure 5B). The proportion of CD8^+^ T cells that were CD103^+^ was much higher than for CD4^+^ T cells, with 50% or more of CD8^+^ T cells expressing CD103 in both LC and TI biopsies (Figure 5B).

It has been reported that Th17 CD4^+^ T cells are selectively reduced in GALT during HIV infection (14, 17, 18) and that this depletion may not be reversed by ART (7, 14, 18). Therefore, we studied the relative proportions of CD4^+^ T cells that expressed CD161, which includes Th17 cells in human GALT (33). However, the results show that there was no significant selective depletion of CD161^+^CD4^+^ T cells in gut biopsy samples from HIV^+^ subjects, with similar percentages compared to HNC (Figure 5C).

Finally, we also used the CD25^+^CD127^dim^ phenotype to identify Tregs (49), although CD25 expression was not always as distinct in the gut biopsy preparations compared to that typically found on Tregs in peripheral blood (Figure S3 in Supplementary Material). Overall, we found a slight increase in the proportion of Tregs in CD4^+^ T cells in both LC and TI biopsies from HIV^+^ subjects, compared to HNC (Figure 5D). Interestingly, the proportion of memory CD4^+^ T cells that were Tregs in TI biopsies from HNC was quite low compared to blood (43, 49), but even with the elevation seen in HIV^+^ subjects, Tregs remained quite a minor subset of CD4^+^ T cells in TI biopsies.

## Discussion

In this study, we aimed to quantify the level of depletion of important subsets of CD4^+^ T cells in GALT biopsies from ART-treated HIV-1-infected subjects, compared to HNC, by accurate counting of single cell suspensions using polychromatic flow cytometry.

We used a novel approach of EpCAM staining of epithelial cells versus CD45^+^ staining of lymphocytes, to optimize detection of CD4^+^ T cells and minimize cell loss during processing. The total numbers of CD4^+^ T cells present in the gut biopsies from the HIV^+^ subjects on ART were at about half the normal level in TI and LC but with no significant difference in RC. There have been differing reports on whether CD4^+^ T lymphocytes are fully reconstituted in GALT following ART (50). Our results suggest that this may depend on which site has been studied, consistent with a recent report comparing different sites of GALT biopsies (51). In a limited number of subjects who commenced ART during PHI, there was no clear difference in CD4 T cell counts compared to the remainder of the cohort studied, who commenced ART during CHI.

Previous studies have mainly used histological enumeration of CD4^+^ and CD8^+^ T cells in the context of HIV infection, and most previous studies have reported depletion of CD4^+^ T cells as a percentage of CD3^+^ lymphocytes (5, 17). We measured the absolute numbers of cells and found a decrease in the CD4:CD8 ratio, consistent with previous results (51).

One of the main aims of this study was to assess eventual changes in the number of Tfh cells in GALT in patients with HIV infection on ART. This is the first study to comprehensively study the Tfh subset of CD4^+^ T cells and the IgA^+^ B cell subset in human gut biopsies during HIV-1 infection. We and others have recently shown dysregulation of Tfh numbers in peripheral lymph nodes and spleen, during either SIV or HIV infections (28–32), including evidence of infection of Tfh (30–32, 52). In all these reports, the proportion of Tfh increased, rather than decreased, compared to other memory CD4^+^ T cells in lymphoid tissue. Nevertheless, based on the descriptions of very extensive CD4^+^ T cell depletion in GALT during primary infection, and also dysfunction of GCs (26), we hypothesized that Tfh were irreversibly lost. However, in treated HIV^+^ subjects, the numbers of Tfh cells in gut biopsies were within the observed normal range.

Consistent with this normal number of Tfh cells, we also found that the number of IgA^+^ B cells was within the normal range in HIV^+^ subjects on ART. The local production of IgA is believed to be one of the main defense mechanisms within the GI tract, yet there have been few studies of the effect of HIV-1 infection on IgA^+^ B cells in GALT. In the study that suggested that GC formation was dramatically affected by PHI (26), longitudinal studies following ART were not performed. One other study also reported a finding of hyperactivity of B cells in GALT during late stage infection, using immunohistochemistry (53), similar to that which has been found in peripheral lymph nodes, and that the hyperactivity was rectified by ART. This normalization even when therapy is commenced in late-stage disease is consistent with our current findings on numbers of IgA^+^ B cells by flow cytometry. Furthermore, the close correlation between Tfh cell counts and IgA^+^ B cell counts is consistent with a very close functional relationship, normalized in HIV^+^ subjects on ART. Further studies should confirm whether luminal concentrations of IgA are also within the normal range.

One limitation of the current study is that we do not have Tfh or IgA^+^ B cell counts during PHI, prior to commencing therapy, and therefore, we cannot determine whether these cells were preserved or restored. Nevertheless, the observed numbers of Tfh cells and the IgA^+^ B cells in GALT after ART suggest that irreversible disruption of the homeostasis of the microbiome and subsequent microbial translocation after ART may not be as severe as currently believed. Previous results, based on immunohistochemistry of biopsies, had suggested disruptions to the epithelial barrier [reviewed in Ref. (10)], with a recent report of excessive proliferation and apoptosis of epithelial cells in CHI (35). We found no difference in the number of epithelial cells between controls and HIV^+^ subjects, and we have been unable to find significant disruption of the epithelial barrier using the technique of *in vivo* confocal endomicroscopy (54).

The main cause of the massive depletion of CD4^+^ T cells from GALT during primary HIV or SIV infection is believed to be high expression of CCR5 on CD4^+^ T cells, as well as activation due to the presence of microbial products (6). However, using an optimized method for staining for CCR5, we found that typically less than half of CD4^+^ T cells in the gut biopsy samples were CCR5^+^ in healthy adult controls, and only a few of these cells expressed markers of activation. We can exclude an effect of enzymatic digestion during the single cell preparation on the detection of either CCR5 or activation markers, since nearly all CD8^+^ T cells were positive for CCR5 in the same preparations, and CD38 and HLA-DR were both present on B cells as expected (data not shown). One previous study showing high levels of CCR5 expression was based on CD45^+^ mononuclear cells and did not distinguish between CD4^+^ and CD8^+^ T cells (20). In that study, a high proportion of CCR5^+^ CD8^+^ T cells may therefore have masked a lower proportion on CD4^+^ T cells.

Also, we know from studies of circulating CD4^+^ T cells that there is an elevation of CCR5^+^ activated CD4^+^ T cells during PHI (55, 56), as well as following vaccinia inoculation (40), and this is consistent with elevated expression of CCR5 on CD4^+^ T cells in GALT once PHI is established (21). Nevertheless, it is believed that under usual steady-state conditions, GALT is normally more anti-inflammatory than proinflammatory (57, 58). Furthermore, it is probable that the majority of CD4^+^ T cells in GALT recirculate, based on their low level of expression of CD103 reported in this study, and on mathematical modeling of CD4^+^ perturbations after large-scale apheresis (59). Conversely, parabiosis experiments in mice demonstrate a slow and incomplete equilibration of CD8^+^ T cells between blood and GALT (60), consistent with our finding of higher expression of CD103 on CD8^+^ T cells. Therefore, taken altogether, it seems unlikely that healthy adults have a preponderance of pre-existing, activated, and resident CCR5^+^CD4^+^ T cells in the GALT, prior to HIV-1 infection.

In our assessments of subsets of CD4^+^ T cells, we found no proportional differences in CD103^+^CD4^+^ T cells, believed to represent intraepithelial/tissue-resident cells (48). Although it has been suggested that preparations of cell suspensions for flow cytometry give a different result for tissue-resident T cells compared to histology (61), this was described in lung tissue and was mainly due to circulating cells within microvasculature in the lung tissue. Our gut biopsy samples were neither significantly contaminated with blood, as indicated by lack of neutrophils and NK cells, nor did they contain visible red cells (data not shown). We rigorously and accurately defined CD4^+^ T cells using polychromatic flow cytometric techniques, excluding possible non-specific staining due to non-lymphoid cells, B cells, or myeloid cells.

Finally, previous studies of subsets of CD4^+^ T cells in gut biopsies during HIV infection have concentrated on the Th17 subset of CD4^+^ T cells, since it is believed they are essential to maintenance of the epithelial cell barrier (10), but we did not find any significant selective effect on CD161^+^CD4^+^ T cells, which are known to include Th17 cells in gut (33). Also, we found only a slight increase in the proportion of Treg cells, which is consistent with our previous results that the majority of Tregs in blood do not express the gut-homing integrins α4 and β7 (62).

In conclusion, the results of this study suggest that, consistent with previous reports (50), ART-treated HIV^+^ subjects may not completely normalize their total CD4^+^ T cell populations in GALT, but their important Tfh/GC/IgA axis of immunity is relatively normal.

## Author Contributions

JZ, MB, GM, KM, NS, and YX performed experiments. MD, KK, AK, and MAB wrote study protocol. MD, GM, DT, MAB, and KK recruited subjects and collated clinical data. JZ, MD, GM, DC, MAB, AK, and KK wrote the manuscript.

## Conflict of Interest Statement

The authors declare that the research was conducted in the absence of any commercial or financial relationships that could be construed as a potential conflict of interest.

## Supplementary Material

Figure S1 **Tfh cells in gut biopsies were identified as CD45RA^−^PD-1^high^CXCR5^+^CD4^+^ T cells (representative flow plot on left) with confirmation of expression of the lineage-specific transcription factor Bcl-6 (right), in TI biopsy from an HIV-negative control**.

Figure S2 **Germinal centre B cells in gut biopsies were identified as CD19^+^CD38^high^ (representative flow plot on left) and CD20^high^Bcl-6^+^ (right), in TI biopsy from an HIV-negative control**.

Figure S3 **Sequential gating strategy to identify T regulatory cells (Tregs) in gut biopsies as CD4^+^CD45RA^−^CD127^dim^CD25^high^**. Representative flow plots are shown for an LC biopsy from an HIV-negative control.

Table S1 **Subject demographics**.
