# Supplementary figures and images for: CD4+ T Follicular Helper and IgA+ B Cell Numbers in Gut Biopsies from HIV-Infected Subjects on Antiretroviral Therapy Are Similar to HIV-Uninfected Individuals
Source: Front Immunol. 2016 Oct 24;7:438. doi: 10.3389/fimmu.2016.00438 (PMC5075890; doi:10.3389/fimmu.2016.00438)

## Supplementary Figure 1

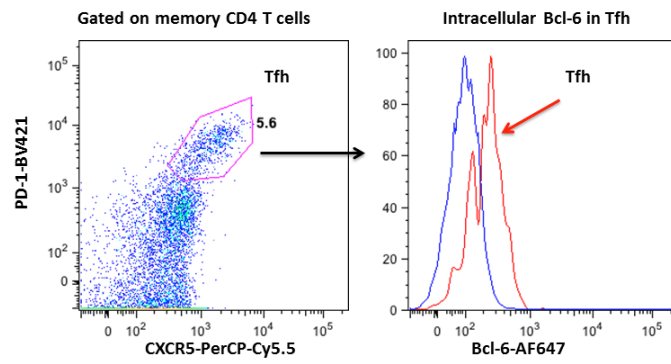

Supplement: Figure S1 — Tfh cells in gut biopsies were identified as CD45RA−PD-1highCXCR5+CD4+ T cells (representative flow plot on left) with confirmation of expression of the lineage-specific transcription factor Bcl-6 (right), in TI biopsy from an HIV-negative control. [file Image_1.PDF]

## Supplementary Figure 2

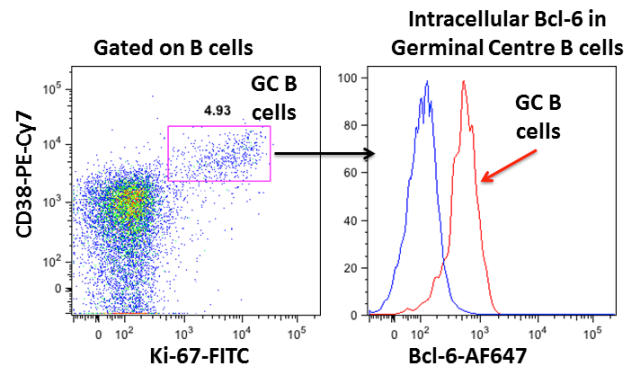

Supplement: Figure S2 — Germinal centre B cells in gut biopsies were identified as CD19+CD38high (representative flow plot on left) and CD20highBcl-6+ (right), in TI biopsy from an HIV-negative control. [file Image_2.PDF]

**Supplementary Figure 3: Gating strategy for Tregs**

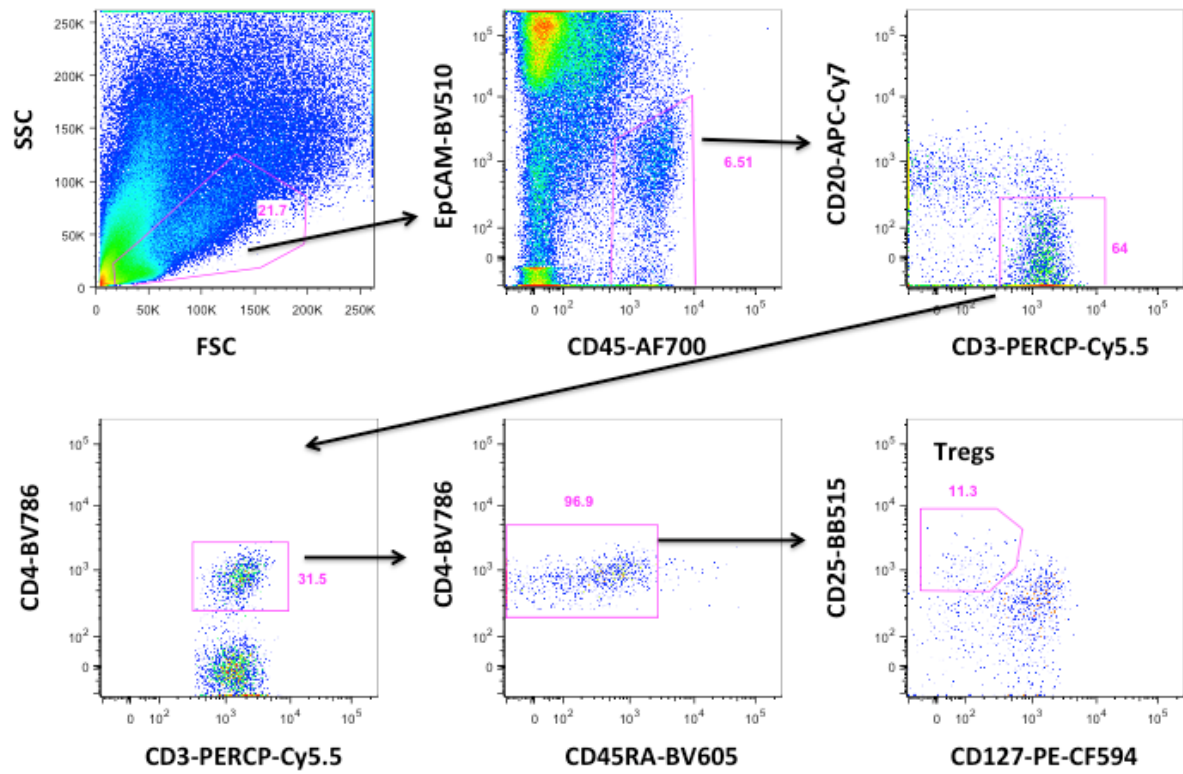

Supplement: Figure S3 — Sequential gating strategy to identify T regulatory cells (Tregs) in gut biopsies as CD4+CD45RA−CD127dimCD25high. Representative flow plots are shown for an LC biopsy from an HIV-negative control. [file Image_3.PDF]
