# Supplementary material for: CD4+ T Follicular Helper and IgA+ B Cell Numbers in Gut Biopsies from HIV-Infected Subjects on Antiretroviral Therapy Are Similar to HIV-Uninfected Individuals
Source: Front Immunol. 2016 Oct 24;7:438. doi: 10.3389/fimmu.2016.00438 (PMC5075890; doi:10.3389/fimmu.2016.00438)
Supplement: Table S1 — Subject demographics. [file Table_1.DOCX]

Supplementary Table 1

| Subject  Group | N | Age (yr)  (median)  (IQR) | Male/  Female | Blood CD4 cell  count/µl  (median)  (IQR) | Blood  CD4:CD8  Ratio  (median)  (IQR) | Viral load  (copies/ml) |
| --- | --- | --- | --- | --- | --- | --- |
| Controls | 22 | 40  (30-54) | 15/7 | N/A | N/A | N/A |
| HIV+ | 23 | 52  (49-55) | 23/0 | 692  (549-773) | 0.94  (0.62-1.46) | <50 |
